# The Role of Brain-Reactive Autoantibodies in Brain Pathology and Cognitive Impairment

**DOI:** 10.3389/fimmu.2017.01101

**Published:** 2017-09-11

**Authors:** Simone Mader, Lior Brimberg, Betty Diamond

**Affiliations:** ^1^The Feinstein Institute for Medical Research, The Center for Autoimmune, Musculoskeletal and Hematopoietic Diseases, Northwell Health System, Manhasset, NY, United States

**Keywords:** autoantibodies, brain, systemic lupus erythematosus, neuromyelitis optica, cognition, blood–brain barrier, maternal antibodies

## Abstract

Antibodies to different brain proteins have been recently found to be associated with an increasing number of different autoimmune diseases. They need to penetrate the blood–brain barrier (BBB) in order to bind antigens within the central nervous system (CNS). They can target either neuronal or non-neuronal antigen and result in damage either by themselves or in synergy with other inflammatory mediators. Antibodies can lead to acute brain pathology, which may be reversible; alternatively, they may trigger irreversible damage that persists even though the antibodies are no longer present. In this review, we will describe two different autoimmune conditions and the role of their antibodies in causing brain pathology. In systemic lupus erythematosus (SLE), patients can have double stranded DNA antibodies that cross react with the neuronal *N*-methyl-d-aspartate receptor (NMDAR), which have been recently linked to neurocognitive dysfunction. In neuromyelitis optica (NMO), antibodies to astrocytic aquaporin-4 (AQP4) are diagnostic of disease. There is emerging evidence that pathogenic T cells also play an important role for the disease pathogenesis in NMO since they infiltrate in the CNS. In order to enable appropriate and less invasive treatment for antibody-mediated diseases, we need to understand the mechanisms of antibody-mediated pathology, the acute and chronic effects of antibody exposure, if the antibodies are produced intrathecally or systemically, their target antigen, and what triggers their production. Emerging data also show that *in utero* exposure to some brain-reactive antibodies, such as those found in SLE, can cause neurodevelopmental impairment since they can penetrate the embryonic BBB. If the antibody exposure occurs at a critical time of development, this can result in irreversible damage of the offspring that persists throughout adulthood.

## Introduction

Over the last several years, many different anti-brain antibodies have been associated with various autoimmune diseases (1). They can be classified as binding either neuronal or non-neuronal antigen and binding extracellular or intracellular antigen (2). Most importantly, the emerging questions are if they can be used for assessment of disease risk, severity, prognosis, and whether they contribute to brain pathology.

Some autoantibodies, such as those present in paraneoplastic disorders of the central nervous system (CNS) or in celiac disease, have been consistently reported to contribute to brain pathology and to cause neurological and cognitive impairment (3–7). For other anti-brain antibodies, such as those present in multiple sclerosis (MS) or narcolepsy, pathogenicity has not been established (8–10).

Given that some autoantibodies can be found also in a subset of healthy individuals (11), it is essential to determine if the antibodies can be used for diagnostic biomarkers of disease or if the autoantibodies are indeed pathogenic. In classic paraneoplastic disorders, where patients have antibodies against intracellular antigens, such as anti-Hu antibodies, it is believed that irreversible killing of neuronal cells is mediated by T cells (12), and the antibodies appear to be a secondary event. In contrast, in some diseases, such as neuromyelitis optica (NMO), the antibodies are pathogenic (13). When pathogenic antibodies enter the CNS, there are two possible outcomes. The pathological consequence of antibody exposure can be reversible. In limbic encephalitis associated with antibodies against cell surface receptors, such as antibodies against extracellular parts of the voltage-gated potassium channel (VGKC) complex, predominantly LGI1, or *N*-methyl-d-aspartate receptor (NMDAR) subunit GluN1, removal of pathogenic antibodies often results in complete remission of severe neuropsychiatric symptoms (12, 14). Alternatively, antibodies may trigger irreversible mechanisms that may continue even when antibody is no longer present in the brain. In patients with systemic lupus erythematosus (SLE) with cognitive dysfunction, pathology can be caused by acute antibody exposure to the NMDA receptor subunits GluN2A/GluN2B and proceed to chronic damage of surviving neurons even when brain-antibodies are no longer present (15).

While brain imaging continues to advance, it remains difficult to study human tissue in those brain diseases. Thus, animal models are needed to study transient and/or permanent tissue injury and to understand which pathology is the consequence of repeated exposure to antibodies and which pathology progresses even in the absence of continued exposure to brain-antibodies. Understanding the contribution of antibodies to disease pathogenesis is essential for the development of efficient and less invasive treatment options and for disease prevention.

## Intrathecal IgG-Synthesis or Systemic Immune Reaction

Brain-reactive antibodies can be produced intrathecally or can be passively transferred from the circulation to the CNS. For example, there is a growing body of evidence that autoantibodies in MS are produced intrathecally and that the presence of antibodies in the cerebrospinal fluid (CSF) is linked to oligoclonal antigen-specific B cells, which infiltrate the impaired blood–brain barrier (BBB) (16, 17). It has been suggested that the antibodies in the CSF of MS patients target ubiquitous intracellular antigens released as cellular debris (18), consequent to neuronal damage and, therefore, constitute a secondary process in disease progression. It may commonly be the case that intrathecal production of antibody is oligoclonal, as the only B cells to take up residence in the CNS may be those that have a B cell receptor for a brain antigen.

Antibodies can also reach the brain from the systemic circulation. Two main questions arise when a disease is caused by circulating anti-brain antibodies. The first question is what triggers their production. One possibility is that the antibodies are triggered by a bacterial or viral infection and cross-react with brain antigens that share structural similarities with the microbial target, a phenomenon defined as molecular mimicry, and was described for example in Sydenham’s chorea (19). Lack of negative selection against CNS antigens as the repertoire of immunocompetent B cells is established might enable activation of B cells with cross-reactivity to brain. In paraneoplastic diseases, antibodies can be produced as a response to a tumor in a non-CNS site, such as lung or ovary, which expresses brain antigens (20).

The second question is how antibodies cross the BBB. BBB endothelial cells express tight junction proteins, which allow only strictly regulated transport into and out of the brain (21). To date, there is limited information available regarding the establishment of the BBB during development; however, there appears to be a time window during which antibodies can penetrate the fetal brain before an intact BBB is established (22). Anti-brain antibodies affecting the developing brain have been suggested to be the cause, for example, in a subset of Autism spectrum disorders, as well as lead to intellectual and cognitive impairments in children born to mothers with SLE (11, 23). In adulthood, certain insults to BBB integrity allow antibodies to penetrate CNS tissue. Different insults to the functionally established BBB lead to different regions of antibody penetration in the CNS. Depending on the location where antibodies gain access to the CNS tissue, various neurological symptoms might occur. Indeed in animal models, region-dependent effects are observed (24, 25). Moreover, circumventricular organs, such as the area postrema, the subfornical organ, and the vascular organ of lamina terminalis, lack tight junction proteins and might be an area for autoantibody entry in some autoimmune diseases (26). Some antibodies might even be able to cause BBB impairment by themselves. In NMO, antibodies to glucose-regulated protein 78 have been associated with BBB disruption (27). In experimental systems, most commonly, the BBB is breached by using either bacterial lipopolysaccharides (LPS) (25), epinephrine (24), and similar agents or by using different pathogenic CNS reactive T cells (28). These manipulations may all result in additional inflammation and make it more difficult to identify the antibody-mediated effect itself.

## Brain Antibodies and Their Pathogenicity

Pathogenicity of brain-reactive antibodies depends on the accessibility of their target epitopes, the density of their presence in tissue and, if required, the presence of effector mechanisms in the brain in sufficient amounts (28). Antibodies from patients are often injected into rodent models and must result in a phenotype similar to the one observed in the human disease to conclude that the antibodies themselves are pathogenic. However, not all patient-derived brain-reactive antibodies bind to rodent tissue; thus, a negative outcome regarding antibody pathogenicity in rodents has to be interpreted cautiously. This is the case in patients with different inflammatory CNS diseases associated with antibodies to myelin oligodendrocyte glycoprotein (MOG), where the majority of human anti-MOG antibodies do not recognize rodent MOG (29). In addition, pathogenicity of brain-reactive antibodies requires breach of the BBB when antibodies are injected systemically into the rodent blood stream. As stated above, insults to the BBB may add confounding factors to the study of antibody pathogenicity and will direct antibody penetration to certain brain regions, which may or may not be those brain regions most often targeted in patients. Alternatively, antibodies can be directly injected into the brain by stereotactic injection, which bypasses the need to breach the BBB. In order to investigate if the antibody results in cognitive impairment, a recognized and sometimes subtle consequence of antibody-mediated pathology, a battery of behavioral assays is performed using *in vivo* models. As more and more brain antibodies are discovered, we need to extend our *in vivo* studies to address whether pathological damage is caused by direct exposure to brain antibodies or if pathology persists even when the antibody is no longer present. Most studies have focused on the effect of acute antibody exposure; only a limited number of studies addressed a possible secondary stage of damage even when the antibody is no longer present in the brain. This secondary stage could be caused through inflammation caused by infiltrating T cells, microglial activation with secretion of proinflammatory cytokines.

For example, in a model of neurocognitive SLE, it is documented that anti-DNA/anti-NMDA receptor antibodies (DNRAbs) lead to persisting neuronal damage even after the antibodies are no longer present (15). It has been recently entertained that the surviving neurons are compromised as a secondary effect mediated by microglia (15). These extended *in vivo* studies are very important for future therapeutic targeting in disease, since removal of antibodies might prevent acute tissue damage, but may not address a subsequent disease phase.

Whereas *in vivo* models to study antibody-mediated brain disease in adults all require a BBB breach, pathogenicity of maternal anti-brain antibodies can be determined without BBB impairment since the fetal BBB allows penetration of antibodies for a period of time (23, 30). Thus, injection of antibodies into pregnant rodents or immunization of rodents with the antigen prior to pregnancy permits a subsequent investigation of the offspring for behavioral impairment and/or histological abnormality. Injecting antibodies into pregnant rodents enables the study of the effect of maternal antibody exposure at one particular time point, whereas immunization with the antigen results in exposure to maternal antibody throughout pregnancy. The binding of maternal anti-brain antibodies to embryonic brain will depend on the expression level of the antigen, which can vary from expression in the adult brain. Furthermore, some antigens exhibit distinct posttranslational modification in the embryonic brain; for example, there may be differences in glycosylation patterns of the antigen (31), which may affect the binding of the antibodies.

## Brain Antibodies and Their Mechanism of Action

Following the proof of a pathogenic effect of brain-reactive autoantibodies, it is of central importance to investigate the pathogenic mechanism(s) in order to develop therapeutic interventions.

In some cases, preexisting inflammation may be required to reveal an antigenic epitope or antibody binding may lead to inflammation giving rise to inflammatory mediators that lead to pathology. Alternatively, complement-dependent cellular cytotoxicity (CDCC) or antibody-dependent cytotoxicity (ADCC) can cause target cell lysis, a possible mechanism of pathogenicity of some autoantibodies (32). Some antibodies can also result in cell death or dysfunction in the absence of inflammatory cell infiltration, CDCC, and ADCC, through altering cell signaling (32). Cell signaling alterations can also activate or impede cellular processes. Finally, antibodies can also cause internalization of membrane receptors, creating functional hypomorphs (2, 32).

The mechanisms of pathogenicity will determine the degree of recovery of brain function. Whereas CDCC and ADCC are more likely to result in irreversible tissue destruction, a pathogenic effect caused by internalization of membrane receptors can be reversed upon removal of antibodies, such as occurs in limbic encephalitis (6). In some autoimmune diseases, therefore, recovery of patients may be linked to the reestablishment of a functionally intact BBB, which prevents further antibody exposure in the CNS. In other autoimmune diseases, brain-antibodies result in a chronic condition, which may be due to constant antibody exposure or to pathology that is no longer dependent on the presence of antibodies (15). Similarly, *in utero* exposure to maternal brain antibodies can cause neurodevelopmental impairments in the offspring that persist throughout adulthood due to irreversible damage at a critical time of development (23, 30).

## Illustrative Examples

In this review, we will describe two autoimmune conditions. First, SLE was discovered to be an autoimmune disease in the 1940s, but antibodies against defined neuronal antigens have been only recently described and linked to neurocognitive dysfunction (33–35). In SLE, pathology may be caused by acute exposure to brain-antibodies, but may persist even upon antibody removal due to irreversible damage and death of neurons and secondary pruning of healthy neurons (15). The role of microglial activation in this secondary disease phase remains to be investigated.

Second, NMO was initially described as a severe variant of MS but due to the discovery of anti-astrocytic antibodies and dramatically different responses to treatment (36, 37) NMO was segregated from MS and defined as a separate disease (38). In order to enable appropriate treatment, we need to understand the reversible and irreversible effects of aquaporin-4 (AQP4)-IgG-mediated tissue damage. In addition, it is important to understand the role of pathogenic T cells for disease initiation as well as for disease progression. Removal of antibodies or blocking of antibody-mediated mechanisms might not be sufficient to address possible disease progression even when the antibody is no longer present.

### Neuron-Directed Antibodies in SLE

Systemic lupus erythematosus is a chronic autoimmune disease that is characterized by inflammation, pain, and tissue damage. SLE can affect any organ, including the brain (39). Since neuropsychiatric manifestations of SLE (NPSLE) are difficult to diagnose due to the diversity of clinical presentations, which include seizures, psychosis, cognitive dysfunction, and more (40), it is difficult to estimate the frequency of neuropsychiatric SLE (NPSLE). Many symptoms, such as headache or demyelination are not unique to NPSLE but can also be found in other autoimmune diseases. Studies claim that as few as 10% to as many as 90% of SLE patients suffer from neuropsychiatric symptoms (41). Cognitive impairment manifested as memory deficit is one of the most commonly observed symptoms in NPSLE patients (42), but is still poorly understood. It may be caused by a variety of mechanisms, both antibody and non-antibody mediated. Hypertension and accelerated atherosclerosis can also lead to cognitive impairment and confound the assessment of diseases-specific mechanisms.

To date, over 100 autoantibodies have been associated with SLE, of which, some associate with neuropsychiatric symptoms (43). Certain autoantibodies, such as anti-ribosomal P, anti-neurofilament, anti-endothelial, anti-Ro, or anti-Smith antibodies have been associated with neuropsychiatric manifestations other than cognitive impairment, whereas anti-neuronal, antiphospholipid, and anti-double stranded DNA (dsDNA) antibodies cross-reactive with the *N*-methyl-d-aspartate receptor (NMDAR) subunits GluN2A or GluN2B (anti-NR2) have been linked to neurocognitive impairment in SLE (44–48). Here, we describe in more detail the contribution of anti-dsDNA–NMDAR antibodies to cognitive impairment in SLE patients.

## dsDNA–NMDAR Cross-Reactive Antibodies Result in Cognitive Impairment in SLE

Anti-double stranded (ds) DNA antibodies are diagnostic of SLE. Previously, our group has shown that some SLE patients harbor anti-dsDNA antibody, which cross-react with a peptide sequence DWEYS present in the extracellular domain of the GluN2A and GluN2B subunits of the NMDAR. This cross-reactivity was first detected using the murine monoclonal anti-DNA antibody R4A. DNA-GluN2 cross-reactive antibodies (DNRAbs) bind to the extracellular part of GluN2 (49). DNRAbs can be detected either by ELISA or by a cell-based assay using human embryonic kidney (HEK) cells expressing the subunits GluN2A or GluN2B in combination with GluN1 (15, 50). They bind preferentially to the active configuration of the NMDAR and enhance the influx of calcium into the cell (51). They are found in approximately 40% of SLE patients (52). It remains to be investigated how the systemically produced DNRAbs gain access to the CNS. It has been suggested that they are able to breach the BBB by themselves (53), or other factors such as cytokines/chemokines or complement activation may be needed.

**Figure 1:**
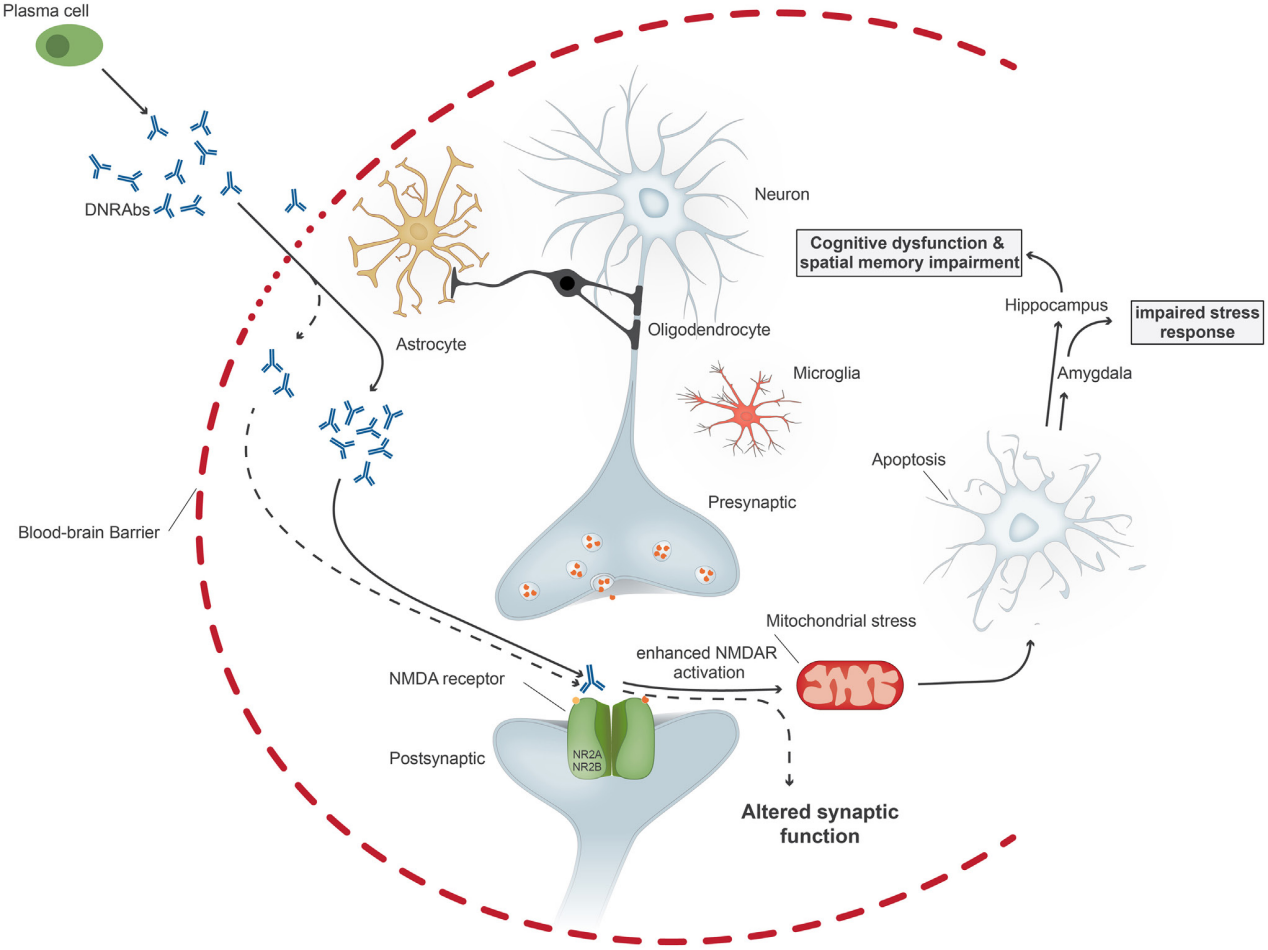
Upon penetration of anti-DNA/anti-NMDA receptor antibodies (DNRAbs) into the central nervous system through the impaired blood–brain barrier, the antibodies trigger two different pathomechanisms dependent on the antibody concentration. At high concentrations (continuous line), the antibodies lead to mitochondrial stress and leads to neuronal apoptosis through receptors containing GluN2A. If the neuronal loss occurs in the hippocampus, the antibody binding results in spatial memory impairment and cognitive dysfunction. Binding of antibodies in the amygdala affects the stress response. At lower concentrations (dotted line), antibody binding results in altered synaptic function.

The pathogenicity of these antibodies was first demonstrated by injecting R4A into mouse brain, leading to apoptosis of neuronal cells. At lower concentrations, the antibody augments NMDAR-mediated synaptic potentials; at higher concentrations, it triggers mitochondrial stress and apoptosis through binding specifically to GluN2A-containing NMDARs (Figure 1). DNRAbs were eluted from the brain of a SLE patient and also caused neuronal apoptosis and cognitive impairment when injected into mice (25). Mice immunized with the DWEYS sequence multimerized on a polylysine backbone (termed MAP-DWEYS) develop DNRAbs, which cause loss of hippocampal neurons after LPS-induced compromise of BBB integrity (54). This occurs in the absence of inflammatory cell infiltration, CDCC, or ADCC. DNRAb-induced neuronal cell death results in cognitive dysfunction and spatial memory impairment associated with structural abnormalities in the surviving pyramidal neurons in the hippocampus (15). The change in spatial memory that occurred after LPS-facilitated DNRAb penetration into the hippocampus is accompanied by expansion in place field size of CA1 place cells in the hippocampus and shortened dendritic processes and spines of surviving hippocampal pyramidal cells (15). Remarkably, the functional and structural changes, which cause alterations in spatial cognition occur at a time when the antibodies are no longer present in the hippocampus and BBB integrity has been restored. Currently, we are investigating the role of microglial activation in the pathology. We believe that there is a two hit model in SLE. Our animal model showed that exposure of neurons to DNRAbs results in neuronal cell death. However, surviving neurons in the hippocampus show structural abnormalities, which are likely to be caused through secondary pruning of the surviving neurons by activated microglial cells (15). In contrast, removal of anti-brain antibodies in limbic encephalitis, which also target the NMDAR, mostly reverses disease symptoms, as these antibodies do not cause cell death.

In the animal model, neuronal cell death can be abolished through administration of the NMDAR antagonist memantine prior to BBB breach by LPS (24). Memantine has no effect on antibody binding, but blocks the triggering of NMDAR activation by DNRAbs.

Studies in patients show that NPSLE is associated with increased levels of GluN2A/GluN2B antibodies in the blood (55), and CSF titers of GluN2A/GluN2B antibodies correlate with the severity of NPSLE (56). Some studies have also associated cognitive impairments in NPSLE with the presence of anti-GluN2A/GluN2B antibodies (57, 58). Certainly, cognitive impairments in NPSLE will not be solely caused by those antibodies; other antibodies or cytokines likely also contribute to cognitive problems in NPSLE (55).

## Maternal DNRAbs are Neurotoxic and have a Gender-Specific Effect

During fetal development, pathogenic antibodies such as DNRAbs can penetrate the embryonic brain before the BBB is functionally established (59). Studies suggest an increased incidence of learning disabilities, fetal loss, and altered sex ratio in children of SLE mothers (60–63). It was, therefore, of interest to investigate the effect of DNRAbs on the fetal brain. We established a mouse model in which pregnant mice expressed DNRAbs throughout gestation (30, 64). Maternal DNRAbs antibodies caused neuronal death in the fetal neocortex and resulted in cortical abnormalities and cognitive impairment in the adult male offspring. In contrast to the cognitive impairment observed in male mice, maternal DNRAbs resulted in increased death of female fetuses, thereby skewing the gender ratio of living offspring (30, 64). We showed that there was no difference in transplacental transfer of the pathogenic antibodies to male or female fetal brain. The gender-dependent effect may be explained by an increased expression of GluN2A in the fetal female brainstem during development compared to male littermates, or to gender-dependent differences in the vulnerability of fetal neurons to GluN2A signaling (64). Neutralization of pathogenic antibodies during pregnancies, perhaps by decoy antigen, may prevent neurodevelopmental impairment.

It should be noted that other antibodies present in SLE patients may affect fetal neurodevelopment. For example, antiphospholipid antibodies can lead to placental problems affecting fetal growth or fetal loss. Moreover, a study suggested that learning disabilities in children born to a mother with SLE were associated with high titers of maternal antiphospholipid antibody (65).

### Astrocyte-Directed Antibodies in NMO

While most anti-brain antibodies target epitopes expressed on neuronal cells, anti-brain antibodies can also be directed to antigens expressed on non-neuronal cells, and thereby cause different brain pathology. In NMO, anti-brain antibodies bind to a protein expressed on astrocytes. NMO is a neurological autoimmune disease that is characterized by the presence of antibodies that bind to the water channel protein AQP4 (66), which is expressed on astrocytic endfeet that surround blood vessels. AQP4 is particularly expressed at the BBB interface. Approximately 80% of NMO patients harbor AQP4 antibodies and the presence of AQP4 antibodies has important diagnostic and prognostic significance (67, 68). These antibodies are conformation dependent and can be detected with highest sensitivity using a cell-based assay with HEK cells expressing AQP4 on their cell surface (67, 69). The presence of AQP4 antibodies differentiates NMO from MS, which have overlapping clinical symptoms, particularly at disease onset. It is of high importance to differentiate MS from NMO since they benefit from different treatment choices (70). Several studies consistently showed that AQP4 antibodies are not present in MS patients or healthy controls and if found they predict development of NMO (68). Thus, AQP4-IgG serostatus has been included in the diagnostic criteria for the disease (38).

Neuromyelitis optica patients have lesions in areas of high AQP4 expression, such as the brain, optic nerve, and spinal cord (71). Histological findings show antibody deposition around blood vessels in the brain of patients (72). The disease primarily presents with astrocyte loss, inflammation with infiltration of granulocytes, macrophages and T cells, deposition of antibodies and complement around blood vessels and, in a later stage of the disease, demyelination, neuronal loss, and scar formation (72, 73). It remains to be investigated how AQP4-IgG that binds to astrocytes can damage oligodendrocytes and how the demyelination observed in NMO occurs. AQP4 antibodies are produced in the systemic circulation of patients and can be found at high serum titers in the CSF (74, 75). It has been suggested that AQP4 antibodies are produced through molecular mimicry to certain microbes (76), a hypothesis, which needs to be further investigated.

Several *in vitro* and *in vivo* models show a pathogenic effect of the AQP4 antibody either by itself, in association with pathogenic T cells, complement or different cytokines and chemokines (28, 77–81) It is possible that AQP4-IgG acts through multiple mechanisms, as suggested by pathological findings showing that, within the same patient, complement deposition is present in some active NMO lesions, while other lesions lack complement deposition (82). In current rodent models, either AQP4-IgG is injected directly into the brain or the BBB is breached prior to antibody injection, often by autoimmune encephalitis (EAE), administering activated autoreactive T cells directed to different CNS antigens (79, 83). In the human disease, we do not know how antibodies enter the brain. It has been suggested that circumventricular organs might be a possible route of entry, supported by findings of NMO lesions in these areas particularly at disease onset (84). Antibodies directed to glucose-regulated protein 78 were recently associated with BBB disruption in NMO and might facilitate penetration of AQP4-IgG antibodies into the CNS (27).

**Figure 2:**
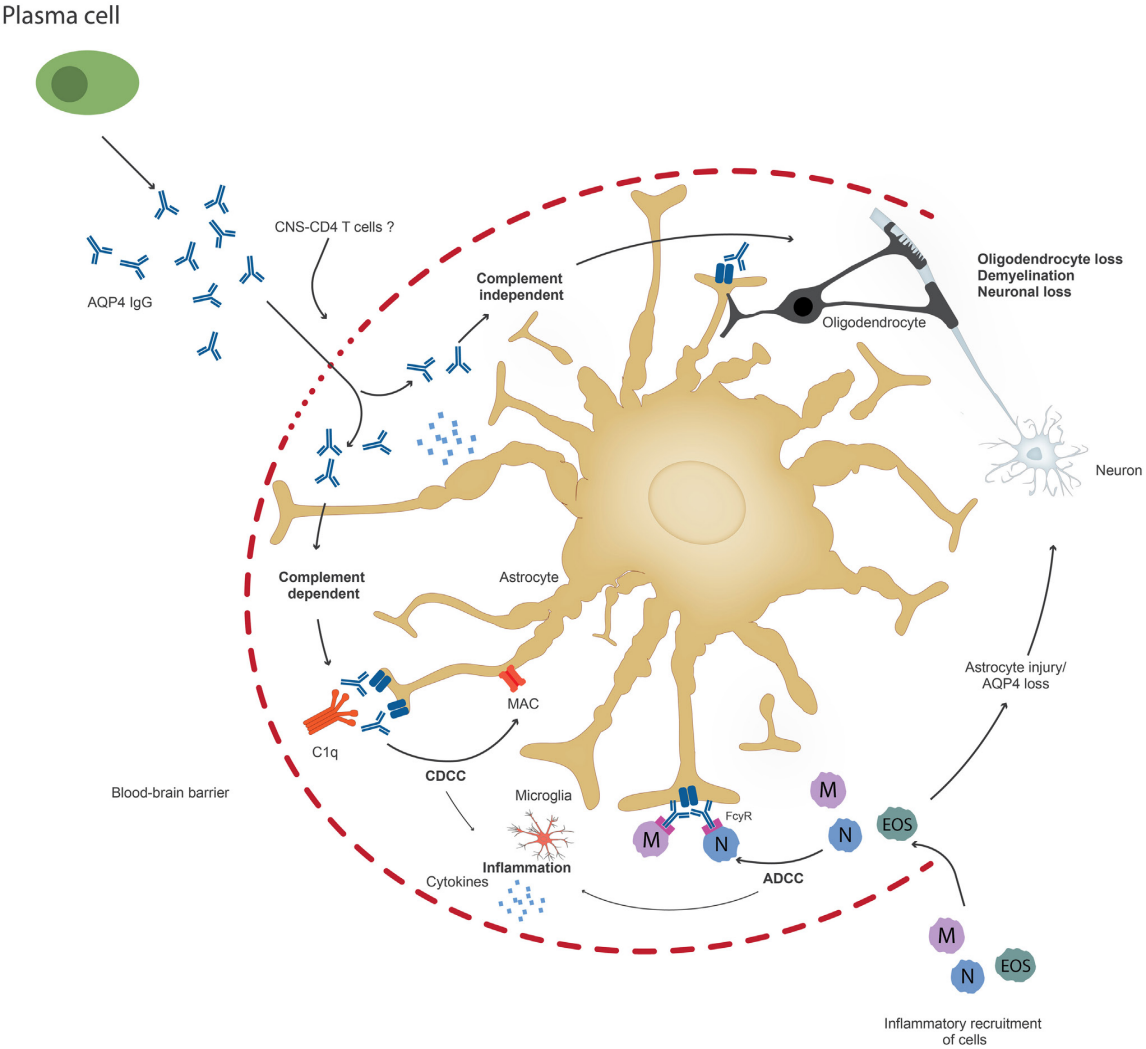
Upon penetration of aquaporin-4 (AQP4) antibodies to the central nervous system, possibly through the help of pathogenic T cells, AQP4 antibodies can lead to astrocyte impairment either through complement-dependent cellular cytotoxicity (CDCC), antibody-dependent cytotoxicity (ADCC), or through downregulation of AQP4. Inflammation might occur prior to or secondary to antibody penetration. Ultimately, oliogdendrocytes are affected resulting in demyelination and neuronal loss.

Once AQP4 antibodies penetrate the brain, they bind to astrocytes and trigger CDCC or ADCC (37) (Figure 2). It has been suggested that these two mechanisms result in astrocyte loss and inflammation and cause or increase BBB damage, which leads to further oligodendrocyte injury and demyelination, which finally results in neuronal loss (37). However, it is also possible that inflammation occurs prior to AQP4 IgG infiltration. It is possible that pathogenic T cells, maybe AQP4 specific or directed to other CNS antigens, are important not only to facilitate antibody production, but also for BBB disruption and may be required for astrocyte and neuronal damage. Current animal models do not closely resemble human patients with respect to the size and location of NMO lesions (13). This discrepancy could be caused by the choice of the target antigen of pathogenic T cells in animal models (85). It is also possible that NMO patients harbor not only AQP4 antibodies but also antibodies to neuronal antigens, which may or may not contribute to disease pathology. More studies are needed that address the role of other antibodies, microglial activation, and proinflammatory cytokine secretion, which could also be responsible for irreversible disease damage observed in NMO, possibly even when the AQP4-IgG antibodies are no longer present in the brain. There is evidence that AQP4-IgG also affects AQP4 function (86).

Cognitive dysfunction has only been recently assessed in NMO patients and needs further investigation (87–89). NMO patients have a different frequency and pattern of cognitive impairment compared to MS patients, suggesting different mechanisms of brain injury (90). Further animal models are needed to study if AQP4 antibodies contribute to cortical neuronal loss and if they can lead to cognitive impairment.

Current studies are trying to develop less invasive treatment options for NMO patients to bypass the highly immunosuppressive treatment (37). One possibility would be to block the AQP4 antibodies or their mode of action. Recently, Eculizumab, a monoclonal antibody that inhibits the classical complement pathway, has been shown to be an effective treatment in an open-label study, suggesting an important role for CDC in NMO (91). However, other effector pathways cannot be ruled out. Thus, it is very important to understand the direct effect of AQP4-IgG on tissue pathology, whether there are other pathogenic effector mechanisms and what processes may be initiated by antibody but continue even when antibody is no longer present. Targeting acute exposure of AQP4-IgG through inhibiting CDCC or/and ADCC might not be sufficient to prevent tissue destruction.

## Maternal AQP4 Antibodies in NMO Pregnancies

Neuromyelitis optica patients have only recently been shown to have an increased frequency of miscarriages (92). Larger follow-up studies are needed to investigate the long-term effect of *in utero* exposure to AQP4-IgG on children of NMO patients, but there are case studies suggesting that maternal AQP4 IgG might result in birth defects (92, 93). Since astrocytes are expressed rather late in development, it is possible that AQP4 is expressed on astrocyte precursor cells during embryonic development.

## Conclusion

Determining the mechanism of action and pathogenicity of several brain-reactive autoantibodies could facilitate more accurate and rapid diagnosis and enable novel treatment options. Here, we describe two examples of autoimmune diseases, which are mediated, at least in part, by autoantibodies and their pathology is well characterized. Both antibodies in those diseases are targeting extracellular antigens on brain cells, either neurons or astrocytes, but differ in their mechanism(s) of action, and hence their pathology.

Anti-DNA/anti-NMDA receptor antibodies in SLE are targeting neurons, resulting in neuronal cell death by enhancing NMDAR activation. Depending on the localization of BBB impairment, DNRAbs result in different neurocognitive or neurobehavioral phenotypes. There is no CNS inflammation following acute exposure to DNRAbs. The sustained, chronic state of neuronal damage secondary to neuronal death that persists after antibody exposure is no longer present in the CNS may reflect either neuron intrinsic effects secondary to antibody exposure or microglial activation.

Brain antibodies in NMO bind to the astrocyte water channel protein AQP4 and result in irreversible astrocyte damage due to CDCC or ADCC. There is increasing evidence that AQP4-IgG can also act by themselves and result in reversible internalization of the AQP4-IgG complex, which is coupled to the excitatory amino acid transporter (EAAT2) endocytosis (94). Different mechanisms might contribute to reversible and irreversible tissue damage of NMO patients [Figure 2, modified from Ref. (37)]. NMO is an example of an antibody-mediated disease where brain pathology of patients shows an inflammatory infiltrate in the CNS, yet, the role of pathogenic T cells in the disease pathogenesis remains to be investigated.

In order to enable appropriate and less invasive treatment, we need to understand the acute and chronic effects of antibody exposure.

## Author Contributions

All authors contributed to writing the review.

## Conflict of Interest Statement

The authors declare that the research was conducted in the absence of any commercial or financial relationships that could be construed as a potential conflict of interest.
